# Inflammatory Factors: Nonobese Pediatric Obstructive Sleep Apnea and Adenotonsillectomy

**DOI:** 10.3390/jcm9041028

**Published:** 2020-04-05

**Authors:** Yu-Shu Huang, Wei-Chih Chin, Christian Guilleminault, Kuo-Chung Chu, Cheng-Hui Lin, Hsueh-Yu Li

**Affiliations:** 1 Department of Child Psychiatry and Sleep Center, Chang Gung Memorial Hospital and College of Medicine, Taoyuan 33305, Taiwan; yushuhuang1212@gmail.com (Y.-S.H.); auaug0327@hotmail.com (W.-C.C.); 2 Stanford University Sleep Medicine Division, Stanford, CA 94063, USA; christianguilleminault3@gmail.com; 3 Department of Information Management National Taipei University of Nursing and Health Science, Taipei 11219, Taiwan; kcchu8992@gmail.com; 4 Department of Craniofacial Research Center, Chang Gung Memorial Hospital and College of Medicine, Taoyuan 33305, Taiwan; 5 Department of Otolaryngology and Sleep Center, Chang Gung Memorial Hospital and College of Medicine, Taoyuan 33305, Taiwan

**Keywords:** inflammation, interleukins, pediatric obstructive-sleep-apnea, adenotonsillectomy

## Abstract

Background: Inflammation is often considered relating to pediatric obstructive sleep apnea (OSA). We conducted a study investigating cytokines, including Il-17 and Il-23, in children with OSA before and after adenotonsillectomy (T&A), compared with controls. Methods: Children with OSA between age 4 and 12 receiving T&A were prospectively followed. Evaluation before and reevaluation six months after the treatment were done, including polysomnography (PSG), blood tests, and questionnaires. Blood samples were obtained to determine the values of high-sensitivity-C-reactive-protein (HS-CRP); tumor-necrosis-factor-alpha (TNF-α); and interleukin (IL)-1, 6, 10, 12, 17, and 23. We compared the results with an age-matched control group. Results: We included 55 OSA children and 32 controls. Children with OSA presented significant improvement after T&A in complaints, signs, apnea hypopnea index (AHI) (*p* < 0.001), mean oxygen desaturation index (*p* < 0.001), and mean oxygen saturation (*p* = 0.010). Upon entering this study, children with OSA had significantly higher cytokine levels than the controls and significant changes in HS-CRP (*p* = 0.013), TNF-α (*p* = 0.057), IL-1β (*p* = 0.022), IL-10 (*p* = 0.035), and IL-17 (*p* = 0.010) after T&A. Children with improved but persistently abnormal AHI did not have all cytokine levels normalized, particularly IL-23 and HS-CRP. Conclusion: Sleep-disordered breathing can persist after T&A and can continue to have a negative inflammatory effect. HS-CRP and IL-23 may serve as blood markers for the persistence of sleep-disordered breathing after T&A.

## 1. Introduction

Adult obstructive sleep apnea (OSA) syndrome has been associated with a number of comorbidities, particularly in obese patients [1,2,3,4,5,6,7,8,9]. Also, previous studies found that inflammation and OSA are strongly related [10,11]. Many of these comorbidities [1,2,3,4,5,6,7,8,9] have been related to the induction of chronic inflammation, which is common with both obesity and OSA [10,11]. Some systemic inflammatory cytokines such as nuclear factor kappa light chain enhancer of activated B cells (NF-κB); C-reactive protein (CRP); tumor necrosis factor-α (TNF-α); Interleukin (IL)-6, IL-8, IL-1α, and IL-1β; and Interferon-γ (IFN-γ) are related to pediatric OSA. A recent review article showed that plasma IL-6 level was significantly reduced after adenotonsillectomy (T&A) in patients with pediatric OSA, especially those with obesity [12]. However, the results of previous studies are still ambiguous.

In children, a decreased upper-airway (UA) size is considered a risk-factor for UA collapse during sleep. Various factors can lead to decreased UA size in children. One of the most common factors is enlarged adenoids and tonsils, which impact nasal air exchange, lead to mouth-breathing, and thus increase the risk of UA collapse during sleep [13]. Since 1978, T&A has been recommended as a treatment for pediatric OSA, especially when OSA is associated with enlarged lymphoid tissue upon clinical evaluation. However, T&A cannot completely improve abnormal breathing during sleep [13,14,15].

Cytokines are considered as having a major role in aiding or triggering inflammation. Previous studies have shown that plasma pro-inflammatory cytokines, such as high density-CRP (HS-CRP), TNF-α, IL-1β, IL-6, and IL-10, were elevated in children with OSA. Besides, the expression ratio of IL-10 and TNF-α was not normalized even after adenotonsillectomy [12]. In our previous study, we reported that HS-CRP, IL-17, and IL-23 were elevated in children with OSA compared with healthy controls [11] and that most of them had tonsil or adenoid hypertrophy. In this study, we wondered whether T&A could be beneficial on inflammatory factors in these patients. Thus, we followed cytokine level changes in patients with both pediatric OSA and enlarged adenoids or tonsils, before and after T&A, and analyzed possible association between cytokine levels and sleep variables.

## 2. Materials and Methods

Due to the complex interaction between different cytokines, first, we investigated the plasma levels of cytokines available for measurement at the time of study. Second, we evaluated the changes in the plasma cytokine levels 6 months after T&A and analyzed the association between apnea hypopnea index (AHI) and cytokines. Last, we tried to find the most effective biomarkers of pediatric OSA.

### 2.1. Participants

We recruited 60 children with snoring and OSA diagnosed according to the International Classification of Sleep Disorders- the third edition (ICSD-3) criteria [16] and 32 gender- and age-matched healthy controls. Five children in the study group were excluded after evaluation because the children or their caregivers refused T&A.

### 2.2. The Inclusion Criteria for Children with OSA

(1) Children with OSA were required to have signs and symptoms of pediatric OSA, and the diagnosis was confirmed by polysomnography (PSG) (AHI > 1 event/hour or respiratory disturbance index (RDI) > 5 events/hour)
(2) They needed to receive evaluation by otolaryngologists and had the diagnosis of adenoid or tonsil hypertrophy.
(3) T&A was indicated after the evaluation. Besides, children and their caregivers were willing for the treatment.
(4) Healthy controls were recruited in the community through contact with school teachers. They were required to have no symptoms of OSA and to have a noninflammatory status (absence of asthma, severe allergies and eczema, or other atopic/autoimmune diseases) as well as yo be in general good health and with normal nocturnal PSG.

Participants with obesity, previous T&A, craniofacial anomalies, neuromuscular diseases, other neurological or psychiatric disorders, the presence of chronic medical problems, or mental retardation (intelligence quotient < 70) were excluded. We also eliminated from the study any children unable to cooperate with blood sampling or PSG. Obesity was defined according to Taiwan’s general public health tables.

This prospective study was approved by the Chang Gung Medical Center IRB (#105-4797C and 201601757A3C501), and the parents or legal guardians of all participants signed the approved consent form.

### 2.3. The Procedures

**Figure 1:**
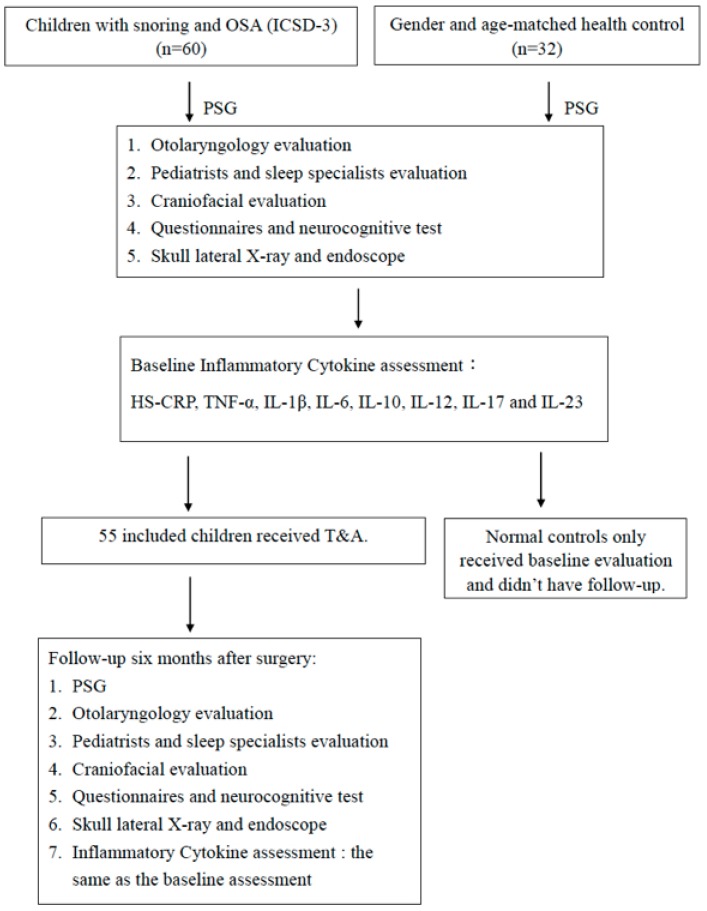
Study flow chart. PSG: polysomnography; T&A: adenotonsillectomy.

(1) Otolaryngologists, craniofacial surgeons, pediatricians, and child psychiatrists assessed each participant’s history of comorbidities and performed routine physical and mental examinations for them (Figure 1).
(2) The participants’ age, gender, height, weight, and all systemic comorbidities were collected.
(3) Tonsillar sizes were graded from 0 to +4 by experts with standardization. The lymphoid tissue was examined using a flexible endoscope in transverse and lateral X-rays. Adenoid tissue was categorized into four grades (from grade 0 = 0%–25% to grade 3 = 75%–100%). The allergic rhinitis testing in allergen-specific IgE (Immuno CAP 100; Phadia, Uppsala, Sweden) has been confirmed based on the duration and persistence of symptoms and on the comorbidities of allergic rhinitis and its asthma classification.
(4) Using nocturnal PSG (A Neural-Virtual BWIII PSG Plus sleep system TM, Fort- Lauderdale. Fl. USA), the following variables were monitored: Electroencephalogram (EEG) (lead 4); Electromyogram (EMG) of eye movement, jaw, and leg; electrocardiogram (lead 1); and position. The respiration was recorded using a nasal pressure sensor mouth thermocouple, the thoracic and abdominal inductive photoelectric pulse band, pendant microphone, diaphragm-intercostal muscle EMG, a pulse oxygen saturation analyzer that can obtain blood oxygen concentration (SaO_2_), finger photoelectric pulse wave, and perform continuous video surveillance. A family member was required to be present during recording through the night. International standards [16,17] were used to measure sleep and wake times, while brain waves were defined by the American Sleep Disorders Association [18]. The definition of apnea and apnea outlined by the American Academy of Sleep Medicine [16] were used to analyze abnormal breathing during sleep. We calculated AHI and RDI (RDI = (respiratory effort-related arousal + hypopneas + apneas) × 60/total sleep time (TST) (in minutes)). The sleep polysomnography tests were scored by certified, and experienced analysts were unaware of the clinical status of each child.
(5) Inflammatory cytokine assessment: blood was coagulated for 30 min after collecting the sample, followed by centrifugal isolation, and the isolated serum was stored at −70 °C. All samples were taken at the same time, which was directly after PSG in the morning. We analyzed the levels of HS-CRP, TNF-α, IL-1β, IL-6, IL-10, IL-12, IL-17, and IL-23 in the serum using highly effective commercial enzyme immunoassay (R&D systems, Minneapolis, MN, USA). Each sample has a copy, and the average was used to statistically analyze the data. The extreme cytokine results in this examination were analyzed using stem and leaf display.
(6) Fifty-five participants with pediatric OSA and adenoid or tonsil hypertrophy received tonsillectomy and/or adenoidectomy (T&A) by sleep surgeons.
(7) The experimental group was evaluated at baseline and 6 months after T&A, while the control group was only evaluated at baseline.

### 2.4. Statistical Analysis

The demographic data are presented as mean ± standard deviation or percentage. When comparing pre- and post-T&A results, we adopted the t-test for repeated measures. When comparing the results of pre-/post-T&A with those of the normal controls, we performed the t-test analysis of variance. Standardized regression was performed to analyze the correlation. All the reported *p*-values are two-tailed with statistical significance set at *p* < 0.05. All statistical data were analyzed with SPSS version 18.

## 3. Results

Demographic data of the OSA group and the control group are shown in Table 1. At baseline (before T&A), compared to the controls, the OSA group had significantly more symptoms of Attention deficit/hyperactivity disorder (ADHD) and enuresis (*p* = 0.001 and 0.036, respectively), and more presence of adenoid or tonsil hypertrophy (both *p* < 0.001). After T&A, the OSA group had significant improvement of enuresis (*p* < 0.001), tonsil hypertrophy (*p* < 0.001), and adenoid hypertrophy (*p* < 0.001) and significantly increased BMI (*p* = 0.001). Compared with the control group, the OSA group still had significantly more ADHD and enuresis symptoms (both *p* < 0.001), more turbinate hypertrophy and allergic rhinitis (both *p* < 0.001), and less tonsil hypertrophy (*p* = 0.04) (though normal controls only had low grade tonsil hypertrophy).

Table 2 showed the results of PSG. At baseline (before T&A), compared with the control group, the results of PSG demonstrated significantly higher AHI, AHI during rapid eye movement period, apnea index (AI), hypopnea index (HI), oxygen desaturation index (ODI), snore index (SI), percentage of sleep efficiency, arousal index, percentage of REM, TST, sleep latency, and mean heart rate in children with OSA (*p* < 0.001, *p* < 0.001, *p* < 0.001, *p* < 0.001, *p* < 0.001, *p* = 0.008, *p* = 0.025, *p* = 0.006, *p* = 0.006, *p* = 0.001, and *p* < 0.001, respectively). After T&A, children with OSA had significantly decreased complaints and improved PSG findings. As shown in Table 2, abnormal breathing indices (AHI, percentage of AHI > 1, AHI during REM, AI, HI, ODI, and SI), arousal index, sleep latency, and mean heart rate significantly decreased, while TST, sleep efficiency, percentage of REM, and mean oxygen saturation significantly increased (Table 2). However, not all children reached AHI ≤ 1 and only 38.2 % had AHI less than 1 event/hour (*p* < 0.001). Many of the breathing variables during sleep still differed from those recorded on the normal controls. Compared with the control group, the OSA group still had significantly higher AHI, AHI during REM, AI, HI, ODI, and SI and significantly more stage N3 and TST (Table 2).

Inflammatory cytokines including HS-CRP and IL-17 (*p* = 0.002 and 0.024) were significantly higher when compared children with OSA with normal healthy controls in our previous study [11]. The results of inflammatory cytokines of this study are showed in Table 3.

(1) Most inflammatory factors demonstrated clear decreases after T&A except IL-23, which was even significantly elevated (23.44 ± 5.57, *p* = 0.003). HS-CRP, IL-1β, IL-10, and IL-17 were significantly decreased (*p* = 0.013, 0.022, 0.035, and 0.010, respectively). Even though the results were not significant for TNF-α (*p* = 0.057), IL-6, and IL-12, a trend toward decreased levels was still observed after T&A. 

(2) After T&A, most plasma cytokines levels of the OSA group were not significantly different from those of the healthy controls, except HS-CRP (*p* = 0.017) and IL-23 (*p* < 0.001). Therefore, we divided the OSA group into two subgroups based on AHI > 1 or ≤ 1 event/hour. 

(3) In the AHI ≤ 1 subgroup, we found that the plasma levels of HS-CRP, IL-1β, IL-10, and IL-17 did not differ significantly from healthy controls, but IL-23 was still significantly higher. In the AHI > 1 group, HS-CRP (*p* = 0.049) and IL-23 (*p* < 0.001) were still significantly higher (Table 3).

IL-23 was significantly higher in the 2 subgroups, and thus, we tried to determine the association between AHI, IL-17, and IL-23. Table 4 showed the percentages of changes in IL-17 and IL-23 among children with OSA whose AHI decreased after T&A. We found that, among these children (n = 46, 83.6%), 67.4% had their IL-23 elevated (n = 31) and 28.3% had IL-17 elevated (n = 13) after T&A. In those whose IL-17 decreased (n = 33, 71.8%), 33.3% had their IL-23 decreased (n = 11), 66.7% had IL-23 elevated (n = 22), and most of them had AHI > 1 (n = 13, 59.1%). In those whose IL-17 increased (n = 13, 28.3%), 69.2% had their IL-23 elevated (n = 9) and most of them had AHI > 1 (n = 7, 77.8%). When considering the association between AHI and IL-23, among those with AHI > 1 after T&A (n = 29), 20 had their IL-23 elevated (68.9%). Among those with elevated IL-23 (n = 31), 20 had AHI > 1 (64.5%). There is a trend that AHI > 1 may associate with elevated IL-23 after T&A. 

We performed a correlation analysis of cytokines and PSG variables and of controlled factors including body mass index, gender, adenoid and tonsil hypertrophy, turbinate hypertrophy, and nasoseptal deviation. We performed the standardized regression test and showed the result in Table 5. We found a positive correlation between HS-CRP with AI and percentage of awake time (r = 0.498 and r = 0.528) and negative correlation with sleep efficiency and TST (r = −0.449 and r = −0.523); a positive correlation between IL-1β and AHI, HI, and percentage of N1 sleep (r = 0.418, r = 0.523, and r = 0.419); a positive correlation between IL-6 and percentage of awake time (r = 0.480); and a negative correlation between IL-10 and 17 with Mean SaO_2_ (r = −0.812 and r = −0.452) (Table 5). There was no significant correlation between TNF-α, IL-23, and PSG variables. The correlation analysis indicated that sleep variables associated with “sleep respiratory disturbances” had a positive correlation with inflammatory factors.

## 4. Discussion

Increase in pro-inflammatory cytokines have been reported in both adult OSA (CRP [19,20], TNF-α, IL-6 [21], and IL-10) and pediatric OSA patients (HS-CRP) [10,11]. In this study, we investigated cytokines in children with both OSA and enlarged adenoids or tonsils and focused on their changes before and after T&A. Similar to our previous finding [11], children with both diagnoses had higher cytokine levels than normal controls (Table 1). Although improvement is always noted, T&A cannot cure all children with OSA [19,20,21,22]. Some children kept having abnormal AHI and cytokine levels after T&A, and the intermittent hypoxia during sleep could still lead to chronic inflammation. It could explain the incomplete resolution of abnormal inflammatory factors in our results. 

Our results showed a possible but nonsignificant association between IL-17 and IL-23 in children with OSA after T&A (Table 4). If IL-17 decreased, IL-23 was more likely to increase. The pro-inflammatory cytokines IL-17 and IL-23 have been emphasized in recent years [11,23,24,25,26,27]. IL-17 is a pro-inflammatory cytokine secreted by T helper 17 cells (Th17) and other cells, including innate immune cells and nonimmune cells [20]. It is referred to as IL-17A andthe IL-17-producing cells secrete IL-17A and IL-17F by stimulation of other cytokines such as IL-1, IL-6, and IL-23, which are secreted by antigen-presenting cells in response to antigen [24,25]. IL-17A and IL-17F form homodimers or heretodimers and bind to inflammation-related cells, such as macrophages, epithelial cells, and endothelial cells [25,26]. The activated cells produce cytokines, including IL-1, IL-6, and TNF-α. These cytokines and inflammatory cells lead to inflammatory responses such as neutrophil recruitment, tissue destruction, and neovascularization. Overreacted responses can result in autoimmune diseases and allergies. Upregulated IL-17A and IL-17F expressions are found during inflammation [27,28], And patients with severe allergies, chronic inflammatory diseases, and autoimmune diseases can have high levels of IL-17 [26,27]. IL-17 and related neutrophilic inflammation in the respiratory system can lead to chronic airway inflammation [26,27,28]. For example, patients with asthma have a high level of IL-17F [29]. IL-17 is associated with adult OSA, and there is an upregulated Th17/T-regulatory (Treg) cell ratio and overexpression of IL-6 and IL-17. Both the imbalance of Th17/Treg and the microenvironment created by oversecreted cytokines can contribute to the development of OSA [30]. In children with OSA, high levels of IL-1β, IL-10, and IL-17A were found in the cytokine profiles obtained from tonsils, indicating inflammation and subsequent T-cell activation [31].

Investigation of IL-17 is valid in evaluating children with sleep-disordered breathing (SDB) [11]. Our study results showed IL-17 significantly decreased after T&A. Compared with the control group, postoperative IL-17 level in both subgroups (AHI > 1 and AHI ≤ 1/hour) had no significant difference (Table 3). It means IL-17 can detect the improvement of AHI but may not be sufficiently sensitive in cases of mild SDB (residual OSA). Residual OSA in children can still impact sleep and some brain functioning involved attention and learning [11]. Nearly all patients’ AHI decreased significantly after T&A and so did their HS-CRP, IL-1β, IL-10, and IL-17. Compared with the controls, there was no significantly difference in TNF-α, IL-1β, IL-6, IL-10, IL-12, and IL-17 after T&A, but IL-23 was significantly elevated after T&A compared with the control group (Table 3). This is an interesting finding. As we know, IL-23 is a cytokine which has immunomodulatory effects [32]. It upregulates memory-cluster-designation-4 (+) T cells, activates the transcription activator, and stimulates the production of interferon-gamma [33,34]. Previous research found that IL-23 can stimulate IL-17’s production and expression [35], and an enhancing effect of IL-17 on TNF-α can mediate IL-23 p19 expression [36]. IL-23 bridges the innate and adaptive arms of the immune response [37], and it is essential for early local immune responses [38]. Interleukin-23 also induces the production of IFN-γ [39,40,41], a vital part of Th1 responses and cell-mediated immunity against intracellular pathogens. IL-23 has a leading role in activating natural killer cells, in enhancing T-cell proliferation, and in regulating antibody production. Considerable evidence supports that increased IL-23 correlates with autoimmune diseases, such as psoriasis, inflammatory bowel disease, rheumatoid arthritis, and multiple sclerosis. IL-23 serves as a maturation factor for Th17 cells, which has just been identified as a primary autoimmune factor. 

We found in two studies that plasma IL-23 was abnormally elevated in patients with OSA [42], associated with enlarged adenoids and tonsils [11,43]. The fact that IL-23 did not return to normal values after T&A is of concern. If the pro-inflammatory interleukin-17, which is predominantly secreted by the T helper 17 cells, is significantly improved, the abnormal upregulation of IL-17 should decrease after T&A. However, IL-23 elevated significantly after T&A despite the clinical improvement. Mouth breathing can be persistent during sleep after T&A and low but still abnormal AHI is commonly noted. It can be the reason of elevated IL-23. In our study, 68.9% children with residual OSA had elevated IL-23 and 64.5% children with elevated IL-23 had residual OSA. Thus, T&A cannot eliminate all breathing events during sleep, and the persistence of mild SDB can still have a negative effect on inflammatory factors.

Such findings should be considered when looking for a blood marker of incompletely resolved SDB following surgery. Besides IL-23, Our results showed that HS-CRP was significantly different only in those with AHI > 1 compared with normal controls (Table 3) and could help to distinguish these patients from those with normal AHI after T&A. However, the many interactions between different cytokines increase the difficulty to interpret our findings. Whether the systemic inflammatory scenario is only due to increased production of pro-inflammatory cytokines or is also influenced by a decrease of anti-inflammatory cytokines remains unclear. We tried to analyze the association between different cytokines, but our small sample size is a limitation. Further investigations of IL-17 and IL-23 on a larger group of children with OSA referred for T&A are necessary to evaluate the potential role of these cytokines as markers of residual pediatric OSA. In addition, it will also be needed to follow the changes of inflammatory cytokines one year after T&A, especially IL-17 and IL-23, because IL-23 has never returned to normal in our study.

A trend toward decreased levels of TNF-α (*p* = 0.057), IL-6, and IL-12 was noted after T&A. Previous studies have indicated a systemic inflammatory state in extremely obese subjects [43], characterized by high levels of TNF-α and IL-12. Such an increase was observed when comparing morbidly obese subjects with normal weight controls. It did not compare morbidly obese individuals versus morbidly obese patients with OSA. The elevated TNF-α and IL-12 in our study could be preferentially related to OSA rather than fat mass and insulin resistance [37,44], since most participants with OSA were not obese and BMI was even lower compared with the control group. A study showed IL-6 was improved after T&A [43], but another study showed no significant change in TNF-α and IL-6 [45]. Both studies evaluated obese children and obesity can influence their results. Though obesity is not an issue in our study, due to small sample size, TNF-α and IL-12 demonstrated only a nonsignificant decrease after T&A. 

In conclusion, T&A can improve pediatric OSA in nonobese children with tonsil/adenoid hypertrophy, shown by the normalization in PSG variables and inflammatory factors. However, T&A cannot cure OSA in some children and mild sleep-disordered breathing persists. Intermittent hypoxia during sleep due to residual OSA can lead to the persistence of chronic inflammation and incomplete resolution of abnormal inflammatory factors. There can be an association between IL-17 and IL-23. If IL-17 decreases, IL-23 is more likely to increase. HS-CRP and IL-23 may serve as blood markers for residual OSA after T&A.

## Figures and Tables

**Table 1:** Demographic characteristics of children with obstructive sleep apnea (OSA) and healthy controls.

	Healthy Control (n = 32)	OSA (Pre-AT) (n = 55)	OSA (Post-AT) (n = 55)	*p* 1 Value (Post-AT vs. Pre-AT)	*p* 2 Value (Post-AT vs. Control)
Number of males (%)	21 (65.6%)	36 (65.5%)	36 (65.5%)	-	0.982
Age (years)	7.02 ± 0.65	7.67 ± 2.64	-	-	0.097
BMI, corrected BMI z score (kg/m^2^) ^a^	17.44 ± 3.08	16.83 ± 4.03	17.67 ± 4.28	0.001 *	0.780
PLM disorder (%)	0 (0%)	1 (1.8%)	1 (1.8%)	-	0.401
Learning disorder (%) ^b^	0 (0%)	3 (5.5%)	3 (5.5%)	-	0.154
ADHD (%) ^b^	2 (6.2%)	29 (52.7%)	20 (36.4%)	0.081	<0.001 *
Enuresis (%) ^b^	4 (12.5%)	24 (43.6%)	10 (18.1%)	<0.001 *	<0.001 *
**Findings of ENT**					
Tonsil hypertrophy(%)	4 (12.5%: gr.2)	50 (90.9%: more than gr. 2)	0	<0.001 *	0.040 *
Adenoid hypertrophy (%) ^c^	3 (9.3%: gr.0)	52 (94.5%: gr.1–3)	0	<0.001 *	0.080
Turbinate hypertrophy (%) ^c^	1 (3.1%: mild)	29 (56.9%)	25 (45.5%)	0.452	<0.001 *
Nasoseptal deviation (%) ^c^	0 (0%)	2 (4.0%)	2 (4.0%)	-	0.237
Allergic rhinitis (%) ^c^	4 (12.5%: mild)	40 (78.4%)	31 (56.4%)	0.073	<0.001 *

* *p* < 0.05. T-test and Chi square test were performed. ADHD: attention deficit/hyperactivity disorder; BMI: body mass index; ENT: ear, nose, and throat; OSA: the OSA group; PLM: periodic limb movement. Pre-AT: before adenotonsillectomy. Post-AT: half a year after adenotonsillectomy; gr.: grade. ^a^ Corrected BMI z score based on the Center for Disease Control (CDC) growth charts. ^b^ Diagnosed according to the criteria of the Diagnostic and Statistical Manual of Mental Disorders (DSM-5). ^c^ Diagnosed by pediatric ENT.

**Table 2:** Comparison of polysomnography findings between groups.

	Healthy Control (n = 32) Mean ± SD	OSA (Pre-AT) (n = 55) Mean ± SD	OSA (Post-AT) (n = 55) Mean ± SD	*p* 1 Value (Post-AT vs. Pre-AT)	*p* 2 Value (Post-AT vs. Control)
AHI > 1 (events/h, n, %)	0 (0%)	55 (100%)	34 (61.8%)	<0.001 *	<0.001 *
AHI (events/h)	0.46 ± 0.28	15.71 ± 22.60	2.98 ± 4.35	<0.001 *	<0.001 *
AHI/REM (events/h)	1.28 ± 1.49	24.75 ± 30.45	5.74 ± 7.52	<0.001 *	<0.001 *
AI (events/h)	0.16 ± 0.18	4.60 ± 8.80	1.25 ± 3.04	0.006 *	<0.001 *
HI	0.39 ± 0.41	11.98 ± 16.20	2.33 ± 4.01	<0.001 *	0.001 *
ODI (events/h)	0.41 ± 0.24	13.52 ± 19.14	3.08 ± 3.82	<0.001 *	<0.001 *
Sleep efficiency (%)	89.90 ± 6.10	84.51 ± 11.77	88.83 ± 7.85	0.025 *	0.494
Awake (%)	6.15 ± 5.13	9.16 ± 11.60	7.26 ± 7.67	0.335	0.446
Arousal Index(events/h)	6.74 ± 3.26	15.83 ± 16.58	8.78 ± 6.63	0.006 *	0.066
REM (%)	18.70 ± 5.44	18.04 ± 5.11	21.00 ± 5.70	0.006 *	0.073
Stage N1 (%)	10.55 ± 6.64	10.45 ± 10.86	7.30 ± 4.31	0.060	0.016
Stage N2 (%)	43.17 ± 9.68	39.29 ± 11.09	40.00 ± 9.61	0.706	0.152
Stage N3 (%)	23.46 ± 12.74	31.82 ± 12.56	31.27 ± 7.96	0.435	0.040 *
TST (mins)	399.40 ± 42.34	382.74 ± 52.22	401.81 ± 21.63	0.009 *	<0.001 *
Sleep latency(mins)	17.90 ± 15.99	25.10 ± 19.96	14.66 ± 11.42	0.001 *	0.324
PLMI (events/h)	0.29 ± 0.28	0.54 ± 1.48	1.23 ± 4.13	0.164	0.135
SI (events/h)	36.26 ± 57.69	208.06 ± 225.2	111.36 ± 166.4	0.008 *	0.006 *
Mean SaO_2_ (%)	96.77 ± 3.77	96.40 ± 2.80	97.45 ± 1.06	0.010 *	0.094
Mean heart rate	77.35 ± 13.79	84.68 ± 11.64	78.17 ± 9.74	<0.001 *	0.773

* *p* < 0.05 (P1: pair T test of post- and pre-AT; P2: independent T test of post-AT and normal controls). SD: standard deviation; Pre-AT: before adenotonsillectomy. Post-AT: half a year after adenotonsillectomy; AHI: apnea hypopnea index; AHI/REM: AHI during REM; AI: apnea index; HI: hypopnea index; ODI: Oxygen Desaturation Index; REM: rapid eye movement; TIB: Time in Bed; SPT: Sleep Period Time; TST: Total Sleep Time; WASO: Wake time after sleep onset; PLMI: Periodic Limb Movement Index; SI: Snore Index; Mean SaO_2_: mean oxygen saturation.

**Table 3:** Comparison of inflammatory cytokines between groups (AHI > 1/hour as the cut point).

	Control (n = 32) Mean ± SD	OSA (Pre-AT) (n = 55) Mean ± SD	OSA (Post-AT) (n = 55) (Mean ± SD)			*p* Value			
			AHI ≤ 1 (38.2%)	AHI > 1 (61.8%)	Total (n = 55)	*p*1 Value Post-AT vs. Pre-AT	*p*2 Value Post-AT (AHI ≤ 1) vs. Control	*p*3 Value Post-AT (AHI > 1) vs. Control	*p*4 Value Post-AT (total) vs. Control
AHI (events/hour)	0.46 ± 0.28	15.71 ± 22.6	0.64 ± 0.28 (n = 21)	4.38 ± 5.01 (n = 34)	2.98 ± 4.35	<0.001 *	0.031 *	<0.001 *	<0.001 *
HS-CRP mg/L	0.42 ± 0.23	3.37 ± 6.04	0.63 ± 0.50 (n = 19)	0.73 ± 0.71 (n = 36)	0.70 ± 0.64	0.013 *	0.153	0.049 *	0.017 *
TNF-α ug/dL	12.62 ± 4.49	13.61 ± 6.37	9.76 ± 3.59 (n = 20)	10.78 ± 7.30 (n = 35)	10.72 ± 6.20	**0.057 ^+^**	0.034 *	0.297	0.157
IL-1β pg/mL	0.42 ± 1.38	1.51 ± 2.25	0.22 ± 0.12 (n = 21)	0.43 ± 0.34 (n = 34)	0.35 ± 0.29	0.022 *	0.422	0.969	0.780
IL-6 pg/mL	1.10 ± 0.92	1.59 ± 1.58	0.94 ± 0.60 (n = 20)	1.39 ± 1.44 (n = 35)	1.22 ± 1.21	0.235	0.487	0.392	0.639
IL-10 pg/ mL	2.10 ± 1.41	2.74 ± 2.94	1.81 ± 1.13 (n = 21)	1.60 ± 1.01 (n = 34)	1.68 ± 1.05	0.035 *	0.463	0.135	0.173
IL-17 pg/mL	10.20 ± 6.36	13.78 ± 7.18	11.85 ± 3.35 (n = 21)	11.02±4.72 (n = 34)	11.33 ± 4.24	0.010 *	0.231	0.560	0.377
IL-12 pg/mL	0.97 ± 0.75 (n = 6)	1.77 ± 1.15	1.26 ± 0.70 (n = 8)	1.18 ± 1.12 (n = 12)	1.21 ± 0.94 (n = 20)	0.229	0.399	0.618	0.416
IL-23 pg/mL	12.29 ± 3.66	20.14 ± 7.08	22.02 ± 3.32 (n = 21)	24.34 ± 6.51 (n = 34)	23.44 ± 5.57	0.003 *	<0.001 *	<0.001 *	<0.001 *

* *p* < 0.05; + *p* < 0.1 (P1: pair T test of post- and pre-AT; P2: independent T test of post-AT and normal control). SD: standard deviation; Pre-AT: before adenotonsillectomy. Post-AT: half a year after adenotonsillectomy; AHI: apnea-hypopnea index; HS-CRP: high-sensitivity C reactive protein; TNF-α: tumor necrosis factor alpha; IL-1β: Interleukins 1 beta; IL-6:Interleukins 6; IL-10:Interleukins 10; IL-17: Interleukins 17; IL-23: Interleukins 23.

**Table 4:** The association between AHI, IL-17, and IL-23 (cutoff point: AHI > 1).

	Change of IL-17	Change of IL-23	AHI Subgroup	Number of Patients(n)
AHI decreased after AT (n = 46)	Increased after AT (n = 13), (28.3%)	elevated after AT (n = 9), (69.2%)	≤1	2
			>1	**7**
		decreased after AT (n = 4), (30.8%)	≤1	1
			>1	3
	decreased after AT (n = 33), (71.8%)	elevated after AT (n = 22), (66.7%)	≤1	9
			>1	**13**
		decreased after AT (n = 11), (33.3%)	≤1	5
			>1	6

AHI: apnea hypopnea index; AT: adenotonsillectomy.

**Table 5:** The association between inflammatory cytokines and PSG parameters.

	HS-CRP	TNF-α	IL-1β	IL-6	IL-10	IL-17	IL-23
AHI, events/h	0.399	−0.013	0.418 *	0.256	−0.001	0.327	−0.120
AHI/REM, events/h	0.308	0.130	0.399	0.267	0.072	0.315	−0.173
AI, events/h	0.498 *	0.038	−0.160	0.174	−0.120	0.097	−0.123
HI, events/h	0.294	−0.001	0.523 *	0.146	0.073	0.184	−0.137
ODI, events/h	0.385	0.018	0.381	0.213	−0.039	0.306	−0.141
Sleep efficiency, %	−0.449 *	−0.222	−0.140	−0.385	−0.199	−0.254	−0.076
Awake, %	0.528 **	0.184	0.184	0.480 *	0.149	0.132	0.143
REM, %	−0.304	−0.226	−0.194	−0.071	0.009	−0.210	−0.182
Stage N1, %	0.344	−0.078	0.419 *	0.334	0.255	0.248	−0.032
Stage N2, %	−0.223	−0.083	−0.204	−0.213	−0.282	−0.361	0.136
Stage N3, %	0.115	0.340	0.093	−0.052	0.231	0.100	−0.047
TST	−0.523 **	−0.154	−0.212	−0.277	−0.188	−0.145	−0.328
Sleep Latency	0.096	0.226	0.014	0.054	0.127	0.147	−0.007
PLM Index	−0.070	−0.208	0.029	−0.052	−0.046	0.003	−0.013
SI	0.374	0.056	−0.109	0.360	−0.018	−0.057	−0.208
Mean SaO_2_, %	0.132	−0.284	0.007	−0.230	−0.812 ***	−0.425 *	0.155

Standardized regression coefficient. Control factors: asthma, allergies, body mass index, gender, tonsil hypertrophy, adenoid hypertrophy, turbinate hypertrophy, and nasoseptal deviation. AHI: apnea hypopnea index; AHI/REM: AHI during REM; AI: apnea index; HI: hypopnea indx; ODI: Oxygen Desaturation Index; REM: rapid eye movement; TIB: Time in Bed; SPT: Sleep Period Time; TST: Total Sleep Time; WASO: Wake time after sleep onset; PLMI: Periodic Limb Movement Index; SI: snoring index; Mean SaO2: mean oxygen saturation. HS-CRP: high-sensitivity C reactive protein; TNF-α: tumor necrosis factor alpha; IL-1β: Interleukins 1 beta; IL-6: Interleukins 6; IL-10: Interleukins 10; IL-17: Interleukins 17; IL-23: Interleukins 23. * *p* < 0.05; ** *p* < 0.01; *** *p* < 0.001.
